# Pseudo-Dipeptide Bearing α,α**-Difluoromethyl Ketone Moiety as Electrophilic Warhead with Activity against Coronaviruses

**DOI:** 10.3390/ijms22031398

**Published:** 2021-01-30

**Authors:** Andrea Citarella, Davide Gentile, Antonio Rescifina, Anna Piperno, Barbara Mognetti, Giorgio Gribaudo, Maria Teresa Sciortino, Wolfgang Holzer, Vittorio Pace, Nicola Micale

**Affiliations:** 1 Department of Chemical, Biological, Pharmaceutical and Environmental Sciences, University of Messina, V.le F. Stagno d’Alcontres 31, 98166 Messina, Italy; acitarella@unime.it (A.C.); apiperno@unime.it (A.P.); mtsciortino@unime.it (M.T.S.); 2 Department of Pharmaceutical Chemistry, University of Vienna, Althanstrasse 14, A-1090 Vienna, Austria; wolfgang.holzer@univie.ac.at; 3 Department of Drug Sciences, University of Catania, V.le A. Doria, 95125 Catania, Italy; davide.gentile@phd.unict.it (D.G.); arescifina@unict.it (A.R.); 4 Department of Life Sciences and Systems Biology, University of Torino, Via Accademia Albertina 13, 10123 Torino, Italy; barbara.mognetti@unito.it (B.M.); giorgio.gribaudo@unito.it (G.G.); 5 Department of Chemistry, University of Torino, Via P. Giuria 7, 10125 Torino, Italy

**Keywords:** coronavirus, cysteine proteases, hCoV-229E, SARS-CoV-2 M^pro^, difluoromethyl ketone

## Abstract

The synthesis of α-fluorinated methyl ketones has always been challenging. New methods based on the homologation chemistry via nucleophilic halocarbenoid transfer, carried out recently in our labs, allowed us to design and synthesize a target-directed dipeptidyl α,α-difluoromethyl ketone (DFMK) **8** as a potential antiviral agent with activity against human coronaviruses. The ability of the newly synthesized compound to inhibit viral replication was evaluated by a viral cytopathic effect (CPE)-based assay performed on MCR5 cells infected with one of the four human coronaviruses associated with respiratory distress, i.e., hCoV-229E, showing antiproliferative activity in the micromolar range (EC_50_ = 12.9 ± 1.22 µM), with a very low cytotoxicity profile (CC_50_ = 170 ± 3.79 µM, 307 ± 11.63 µM, and 174 ± 7.6 µM for A549, human embryonic lung fibroblasts (HELFs), and MRC5 cells, respectively). Docking and molecular dynamics simulations studies indicated that **8** efficaciously binds to the intended target hCoV-229E main protease (M^pro^). Moreover, due to the high similarity between hCoV-229E M^pro^ and SARS-CoV-2 M^pro^, we also performed the in silico analysis towards the second target, which showed results comparable to those obtained for hCoV-229E M^pro^ and promising in terms of energy of binding and docking pose.

## 1. Introduction

In the last few decades, the recurring occurrence of viral pandemics worldwide, i.e., those caused by coronaviruses SARS-CoV (2003), MERS-CoV (2012), and SARS-CoV-2 (2019), indicated that outbreaks of emerging viruses are a severe threat for public health [1]. The current therapeutic arsenal to counter viral infections is confined to drugs that can be deployed against few viruses, mostly producing persistent infections, e.g., human immunodeficiency virus (HIV) [2], hepatitis B virus (HBV), hepatitis C virus (HCV) [3,4], some herpes viruses [5] and influenza virus. However, no specific antivirals have been approved for coronaviruses, with the sole exception of remdesivir (Veklury^®^), a repurposed broad-spectrum RNA-dependent RNA polymerase inhibitor which has been recently approved against SARS-CoV-2, the causative agent of COVID-19 [6,7,8]. Therefore, identifying new direct-acting antiviral agents with potent activity, non-overlapping resistance profiles, limited drug interactions, and minimal adverse effects is urgently needed to treat viral diseases, including COVID-19 [9] effectively.

**Figure 1:**
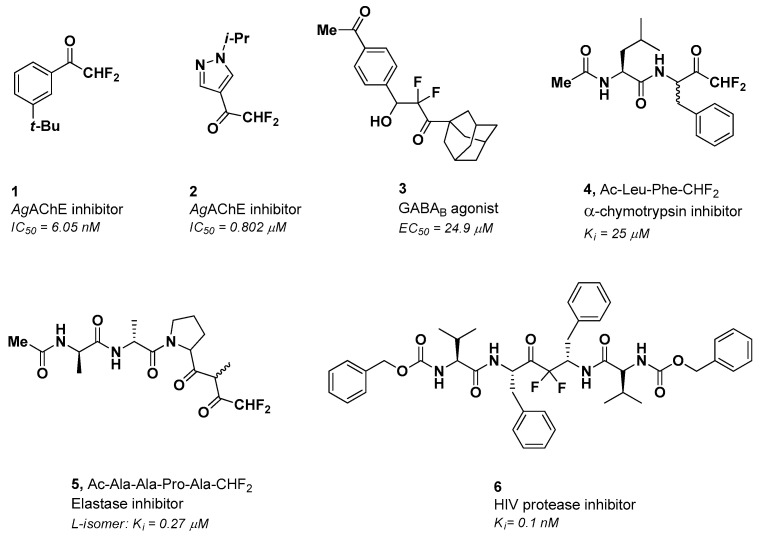
Selected examples of difluoromethyl ketones (DFMKs) with relevant biological properties. *Ag*AChE = *Anopheles gambiae* acetylcholinesterase; GABA_B_ = γ-aminobutyric acid type B.

Part of our ongoing research is aimed at the discovery of small molecules endowed with an “electrophilic warhead” able to inactivate parasitic cysteine proteases by the formation of a reversible or irreversible covalent bond with the catalytic residue of the intended targets [9,10,11,12]. Peptide-based α-fluorinated ketones have long been considered compelling substrates in the drug discovery area to develop selective enzyme inhibitors and activity-based probes [13]. The α-fluorination of the methyl ketone functional group profoundly modifies the physicochemical properties of the related substrate and the reactivity of its carbonyl group [14]. The presence of one or more fluorine atoms in the terminal α position of a methyl ketone moiety enhances the electrophilicity of the carbonyl group, and the resulting fluorinated compound becomes more susceptible to undergo a nucleophilic attack by the catalytic residues of proteolytic enzymes, leading to a slow-binding competitive inhibition of the target by forming hemi(thio)ketal adducts [15,16,17]. Among the three-terminal fluoromethyl groups (-CH_2_F, -CHF_2_, and -CF_3_) next to the carbonyl, -CHF_2_ is the most interesting and yet least explored [18]. This difluorinated group exerts a substantial electron-withdrawing effect on the adjacent carbonyl, making it more reactive and acting as a weakly acidic and lipophilic hydrogen bond donor. It could be considered a valid bioisostere of the hydroxyl or thiol group [19,20,21,22]. To date, a considerable number of α,α-difluoromethyl ketones (DFMKs) have been prepared and evaluated mostly as protease inhibitors (Figure 1). Non-peptidic DFMKs **1** and **2** have been proposed as reversible covalent inhibitors of the *Anopheles gambiae* acetylcholinesterase (*Ag*AChE) [23], whereas compound 3 was investigated as an agonist of the γ-aminobutyric acid type B (GABA_B_) receptor [24]. Compounds **4** and **5** are the only two examples of peptidic DFMKs reported in the literature so far [25,26]. They showed inhibitory activity against bovine pancreatic α-chymotrypsin and porcine pancreatic elastase. Quaternary DFMK **6** instead was investigated as an HIV-1 protease inhibitor [27].

**Figure 2:**
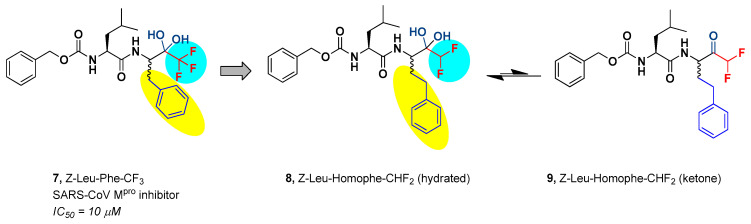
Dipeptidyl TFMK **7** and Z-Leu-Homophe-CHF_2_ in its hydrated form **8** and ketone form **9**. The different fluorinated groups are marked in red; the variable carbonyl group and the P_1_ side chain are marked in blue; chemical variations between lead compound and newly synthesized compound are highlighted in light blue (fluorinated group) and yellow color (P_1_ side chain).

On the other hand, both peptidic and non-peptidic trifluoromethyl ketones (TFMKs) have been widely investigated as protease inhibitors. Besides, a considerable number of biologically active peptidic structures bearing the *C*-terminal -COCF_3_ electrophilic warhead have been synthesized [13,28]. In particular, the dipeptidyl TFMK **7** (drawn in the predominant hydrated form; Figure 2) was evaluated against SARS-CoV M^pro^ in vitro, and a good inhibitor activity was detected [29]. However, compound 7 and its derivatives were not investigated further.

Taking 7 as reference and considering the topicality of the current health emergency, the interest of our ongoing research program aimed at the discovery of new antiviral agents [30,31,32] shifted on the synthesis of the pseudo-dipeptide Z-Leu-Homophe-CHF_2_ (8) and on the evaluation of its anti-coronavirus profile. Z-Leu-Homophe-CHF_2_ retains the overall chemical backbone of the SARS-CoV M^pro^ inhibitor 7, with the exception of the *C*-terminal electrophilic warhead (i.e., DMK in place of TMK) and the Phe residue at the P_1_ site that was replaced by its superior homolog Homophe (Figure 2).

## 2. Results and Discussion

### 2.1. Chemistry

The preparation of the targeted α,α-difluoromethyl ketone was achieved, levering on recent chemistry developed by the Pace’s research group, dealing with the highly chemoselective delivery of α-substituted methyl-type carbanions to competent electrophilic platforms [33,34,35,36,37,38,39,40,41,42,43,44,45,46,47]. In particular, such C1 synthons proved to be excellent nucleophilic elements for acylating Weinreb amides, as documented in extensive work focusing on the access to α-halogenated ketones, inter alia. At the outset of our synthetic plan, we were cognizant of the difficulties of applying such chemistry to complex peptidyl manifolds. However, the success of the tactic observed in the chemoenzymatic synthesis of Nelfinavir that we reported in 2018 made us confident on the feasibility of such an approach [47].

**Scheme 1:**
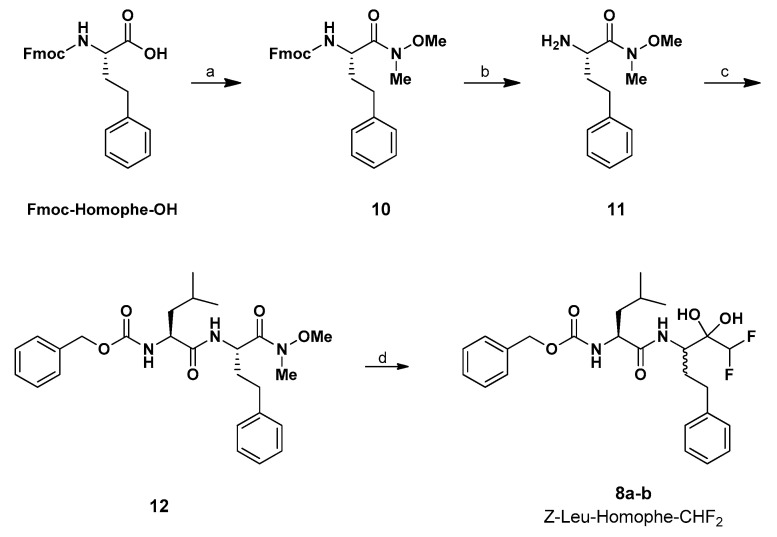
Synthesis of Z-Leu-Homophe-CHF_2_ (**8a**–**b**). Reagents and conditions: (a) CDI (1,1′-carbonyldiimidazole), DMHA (*N,O*-dimethylhydroxylamine hydrochloride), dry CPME (cyclopentyl methyl ether), 0 °C to rt, overnight. (b) Piperidine, dry DMF (*N,N*-dimethylformamide), 24 h, rt. (c) Z-Leu-OH, EDCI, HOBt, dry DMF 0 °C to rt, overnight. (d) TMSCHF_2_, *t*-PentOK, dry THF (tetrahydrofuran), 0 °C, 4 h.

The chemoselective synthesis of the dipeptidyl α,α-difluoromethyl ketone **8** (Scheme 1) consists of a four-step synthetic procedure, starting from the commercially available Fmoc-Homophe-OH, which was converted into the corresponding Weinreb amide **10** with HN(Me)(OMe) HCl (DMHA)**.** Then, the Fmoc group was removed through piperidine treatment and, the resulting free-amine **11** underwent a standard EDCI/HOBt coupling reaction with Z-Leu-OH to afford **12** in 65% overall yield. Finally, it was converted into **8** via the interrupted homologation with a formal CHF_2_ transfer agent, generated upon treatment of the commercially available TMSCHF_2_ with potassium *tert*-pentoxide (i.e., *tert*-amylate) as the activating factor [43].

Pleasingly, the difluoromethylation proceeded under full chemo control, leaving untouched the additional sensitive elements present (amides and carbamate). An excess of potassium *tert*-amylate was used (compared to TMSCHF_2_) to ensure the deprotonation of the acidic NH groups of the amide linkages. Under these conditions, the desired α,α-difluoroketone was prepared in excellent yield (87%) as an inseparable mixture of two diastereomers (**8a**–**b**). Structurally, the carbonyl group was present in the hydrate form (*gem*-diol) in agreement with literature precedents for distinct (poly)α-fluoroketones [18,48,49,50].

NMR spectroscopic data unambiguously supported the presence of the hydrated form of compound **8** as a mixture of two diastereomers (3:1 ratio). The ^1^H NMR spectrum of **8a**–**b** in methanol-*d*_4_ exhibited the characteristic signal ascribed to the -CHF_2_ group (5.69 ppm major isomer; 5.76 ppm minor isomer, ^2^*J*_H,F_ = 54.4 Hz) (see Figure S1). As expected, under the experimental conditions, the compound underwent the partial racemization at α position of -COCHF_2_. The ^13^C NMR spectrum did not show any carbonyl signal when performed in methanol-*d*_4_; a peak at 96.8 ppm corresponding to the quaternary carbon of the hydrated ketone form was observed instead (see Figure S2). In markedly non-polar solvents such as benzene-*d*_6_, the equilibrium is slightly shifted toward the ketone form **9a**–**b**, as revealed by the carbonyl signal at 197.3/197.2 ppm in ^13^C NMR spectrum (See Figure S3). HRMS spectrum displays the peak ascribed to the hydrated form as sodiated species at 501.2176 *m/z* [M + Na]^+^ and the peak ascribed to the ketone form as sodiated species at 483.2065 *m/z* [M + Na]^+^ (see Figure S4). These experimental data suggest that the equilibrium hydrated form/carbonyl form is highly solvent-dependent.

### 2.2. Biological Evaluation

**Figure 3:**
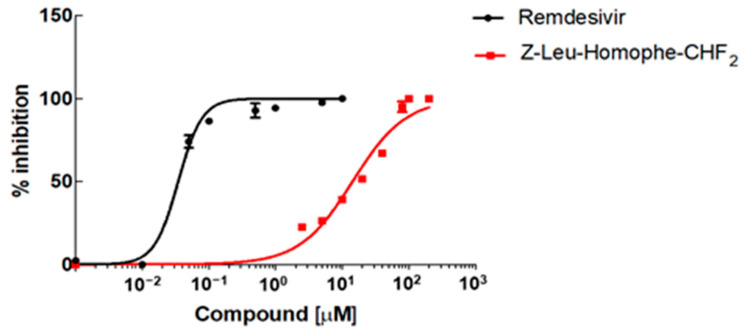
Antiviral activity of Z-Leu-Homophe-CHF_2_ and remdesivir against hCoV-229E. MRC5 cell monolayers were infected with hCoV-229E (100 PFU/well) and treated with increasing concentrations of either Z-Leu-Homophe-CHF_2_ (0–200 µM) or remdesivir (0–10 µM) during virus adsorption and throughout the experiment. At 72 h p.i., cell viability was measured using the CellTiter-Glo luminescent assay. The data shown represent means ± SD (error bars) of two independent experiments performed in triplicate.

The ability of Z-Leu-Homophe-CHF_2_ to inhibit the replication of hCoV-229E, one of the four endemic human coronaviruses and a common cause of upper respiratory tract infections [51], was investigated by a CPE-based assay in normal human lung fibroblasts. To this end, MRC5 cells were infected at a low multiplicity of infection (MOI) to allow for multicycle replication. At an extended endpoint measurement (72 h post-infection, p.i.), a cell viability assay was used as a surrogate of CPE to measure inhibition of hCoV-229E replication [52]. As shown in Figure 3, significant concentration-dependent inhibition of the replication of hCoV-229 was observed in MRC5 cells treated with Z-Leu-Homophe-CHF_2_. The EC_50_ and EC_90_ were 12.9 ± 1.22 µM and 38.6 ± 0.67 µM, respectively. As expected, remdesivir, used as a control of a reference anti-CoV agent, inhibited hCoV-229 replication in the low nanomolar range (EC_50_ 0.034 ± 0.0025 µM, CC_50_ >10 µM), as previously observed in the same type of antiviral assay [52]. Then, the Z-Leu-Homophe-CHF_2_ cytotoxicity was evaluated in different types of uninfected cells, such as the lung fibroblasts MRC5 and human embryonic lung fibroblasts (HELFs), and the lung epithelial cells A549 (Figure S5). The cytotoxic concentrations (CC_50_) were 170 ± 3.79 µM, 307 ± 11.63 µM, and 174 ± 7.6 µM for A549, HELFs, and MRC5 cells, respectively, thus indicating that the antiviral activity of Z-Leu-Homophe-CHF_2_ against hCoV-229E was not due to the cytotoxicity of the target cells themselves.

### 2.3. Computational

**Figure 4:**
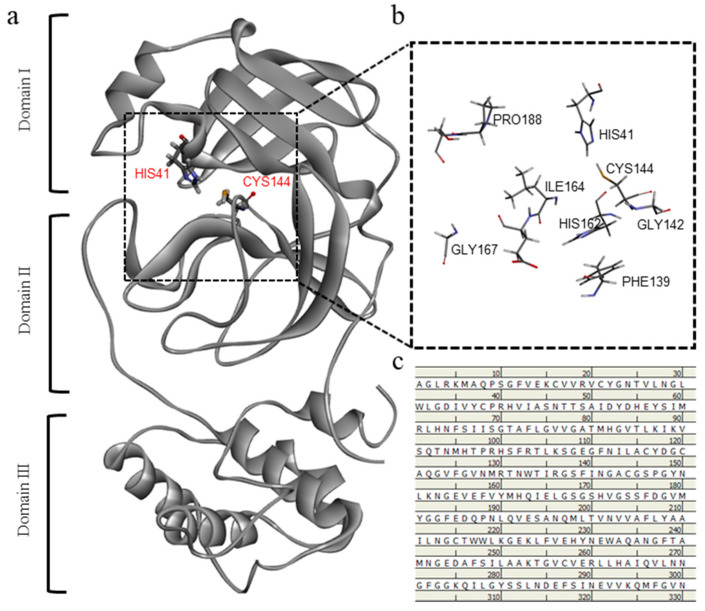
(**a**) Cartoon representation of one protomer of the hCoV-229E M^pro^ (PDB ID: 1P9S). (**b**) Amino acid residues of the catalytic site are indicated as stick. (**c**) The amino acid sequences of M^pro^.

The 2.37 Å crystal structure resolution (PDB ID: 1P9U) of hCoV-229E M^pro^ [53] shows that the protein comprises three domains (Figure 4a). Domains I and II (residues 8 to 99 and 100 to 183, respectively) (Figure 4c). have a high similarity with the structure of the 3C proteinases of picornavirus and chymotrypsin. The substrate-binding site is located in an empty area between these two domains. A long helix loop (residues 184 to 199) connects domain II to the *C*-terminal domain (domain III, residues 200 to 300). This globular cluster of five helices is responsible for the catalytic activity of M^pro^ [54]. hCoV M^pro^ forms a dimer (the contact interface is mainly located between the domain II of monomer A and the *N*-terminal residues of monomer B and has an area of ~1300 Å^2^) with the two monomers oriented in the opposite direction to each other. In the M^pro^ dimer, the N-terminal amino acid residues are in close contact between domains II and III of the parent monomer and domain II of the other monomer, creating specific interactions suitable for binding this portion with high affinity after autocleavage. This mechanism would allow the catalytic site to bind other cleavage sites in the polyprotein. Cys144 and His41 (Figure 4b), in the active site of hCoV-229E M^pro^ to form a catalytic dyad within the S1’ pocket [53].

**Figure 5:**
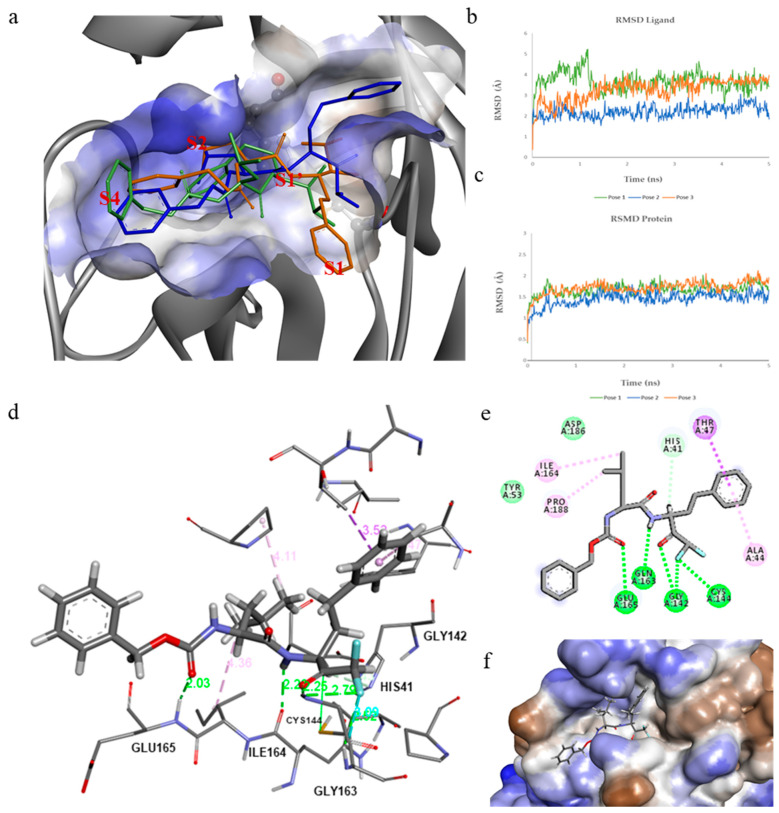
(**a**) Results from molecular docking and their overlap in the hCoV-229E M^pro^ binding site: pose1 (green), pose2 (blue), and pose3 (orange). The binding site of hCoV-229E M^pro^ is represented as a hydrophobic surface (hydrophobic: brown, hydrophilic: blue, neutral: white). (**b**) Time evolution of RMSD of complex with ligand. (**c**) Time evolution of RMSD of backbone atoms of M^pro^ relative to their respective minimized structure and the heavy atoms of the poses. (**d**) Detailed interactions of pose2 of Z-Leu-Homophe-CHF_2_ inside the hCoV-229E M^pro^ binding site. Hydrogen bonds are represented as green dashed lines and hydrophobic interactions with pink dashed lines. (**e**) 2D Interaction inside the binding pocket of the protein. (**f**) Hydrophobic surface of hCoV-229E M^pro^ in complex with Z-Leu-Homophe-CHF_2_.

Molecular modeling studies were conducted to identify and evaluate the receptor/ligand recognition’s key molecular interactions. Molecular docking was performed using the crystal structure of hCoV-229E M^pro^ (PDB ID: 1P9U). The poses presenting the difluoro carbonyl group in the vicinity of the His41/Cys144 catalytic dyad were selected for further stability studies. All three selected poses show a different orientation of the phenethyl group and the isobutyl group (Figure 5a) within the binding site. In fact, in pose 1, the phenethyl group is inserted inside the S2 sub-pocket, while in pose 2 the phenethyl is arranged in the region overlying the S1’ pocket and the isobutyl group occupies the S2 sub-pocket. Pose 3 reverses the positions of the two groups with respect to pose 1. In fact, the phenethyl group is incorporated inside the S1 sub-pocket and the isobutyl group inside the S2 pocket. The only difference between pose 2 and pose 3 lies in the position of the phenethyl group.

To verify the reliability of the docking results, molecular dynamic (MD) simulations were performed based on the results of the molecular docking. The first three Z-Leu-Homophe-CHF_2_ docking poses were evaluated in complex with hCoV-229E M^pro^ for 5 ns of MD simulation. To explore the dynamic stability of both systems and to ensure the rationality of the sampling method, progression of the root mean square deviations (RMSD) from the starting structure was analyzed (Figure 5b). The superposition of the coordinates of each complex structure in a trajectory onto the initial structure allowed us to analyze the RMSD of ligand and protein in complex. The RMSD graph of the ligand (Figure 5c) indicates that the conformations of the complex in pose 1 reach equilibrium around 1.5 ns, while in pose 2 and pose 3, after 0.05 ns and 0.15 ns, respectively. Laying 2 provided a lower RMSD than the starting structure (2.11 Å), and the conformations oscillate on average around 1.3 Å. The protein structure in complex with the ligand in pose 2 also shows a more stable RMSD during MD simulation, reaching equilibrium after 1.5 ns.

Consequently, we focused on pose 2 for further study. Indeed, it has been previously shown that the S2 sub-pocket in the human coronavirus hCoV-229E M^pro^ due to the limited size of the S2 pocket, the benzyl group (in the studied inhibitors) cannot enter deeply into this site, also due to the low plasticity of the pocket [55]. This could explain the better stability of the protein complex in pose 2.

Pose 2 at the binding site of hCoV-229E M^pro^ forms more hydrogen bonds, with the main residue chain in the substrate-binding pocket, which also helps to lock the inhibitor within the media binding pocket by establishing H bonds with Gln163 and Glu165 at 2.03 Å and 2.36 Å, respectively. The isobutyl group inserts itself deeply inside the S2 hydrophobic pocket, stabilizing from hydrophobic interactions with Ile164 and Pro168, with the phenethyl portion by the *π*-CH bond with Thr47 and *π*-alkyl with Ala44. The α,α-difluoromethyl ketone group is located at 3.46 Å from Cys144, stabilized by the H bond with Gln163 at 2.25 A. The H bonds between the fluorine atom and Gly142 and Cys144 at 2.59 Å and 2.52 Å could contribute together with Gln163 to stabilize the transition state during the nucleophilic attack on the carbonyl group. The large benzyl group extends into the S4 site, leaving this region of the ligand exposed to the solvent.

**Figure 6:**
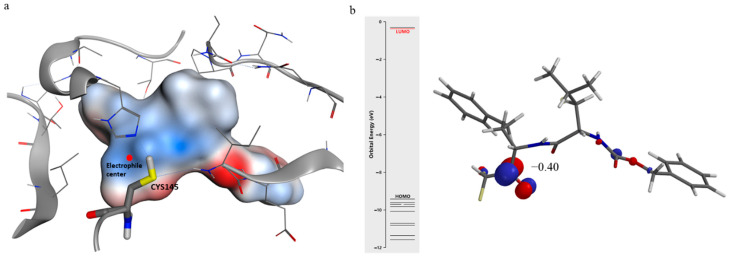
(**a**) The electrostatic potential map (generated in the semiempirical method PM6) of the ligand in the docked pose within the active site of hCoV-229E M^pro^. The positive, negative, and zero electrostatic potential areas of the complex system were represented by red, blue, and white colors, respectively. (**b**) Lowest unoccupied molecular orbital (LUMO) profile of the Z-Leu-Homophe-CHF_2_.

The lowest unoccupied molecular orbital (LUMO) and molecular electrostatic potential (MEP) were performed (Figure 6), with semiempirical PM6 methods, to locate the atom most favorable to the nucleophilic attack by the thiolate ion.

The carbonyl group of difluoromethyl ketone shows a LUMO density and a positive electrostatic potential, which are fundamental for the mechanism of the reaction. The LUMO delineates the areas most lacking in electrons, therefore subject to nucleophilic attack by the thiolate anion.

A 100 ns MD simulation was performed to analyze, in greater detail, any changes in the protein-ligand complex. The protein structure and the ligand reached equilibrium after 30 ns and a very similar average RMSD, 2.1 Å, and 2.3 Å for ligand and protein, respectively (see Figure S6). The flexibility of the different residues of hCoV-229E M^pro^, by calculating the root mean square oscillations (RMSFs) of Cα atoms, was analyzed. A relatively higher RMSF value is obtained around residue 50 (domain I), while more excellent stability occurs in domain II.

Furthermore, to understand the effect of Z-Leu-Homophe-CHF_2_ binding on the internal dynamics of hCoV-229E M^pro^, the dynamic cross-correlation matrix (DCCM) was calculated by using the coordinates of Cα atoms from the trajectories. Domain I (residues 8–99) displays correlated motions, while domain II (residues 100–183) shows highly anti-correlated movements. However, domain III (residues 201–300) has a lower strength of anti-correlation motions relative to domain I. Furthermore, it is evident from (See Figure S6) that the inhibitor binding increases the strength of anti-correlated motions in the region of domain II, which indicates the residual motion of domain III. Overall, the binding of Z-Leu-Homophe-CHF_2_ with M^pro^ creates a stable environment near the binding cavity.

Given the high similarity between hCoV-229E M^pro^ and SARS-CoV-2 M^pro^, we decided to investigate the in silico affinity between Z-Leu-Homophe-CHF_2_ with SARS-CoV-2 M^pro^. However, there are many common features shared between the two types of proteases, particularly their almost absolute requirement for Gln in the S1 position of the substrate and space for only small amino acid residues as a common target for the design of broad-spectrum antiviral compounds. Since there is no human protease with a specificity for the Gln residue at the substrate’s catalytic cleavage site, the interest of this viral target increases, as it increases the possibility that the inhibitors developed will not show host cell toxicity.

**Figure 7:**
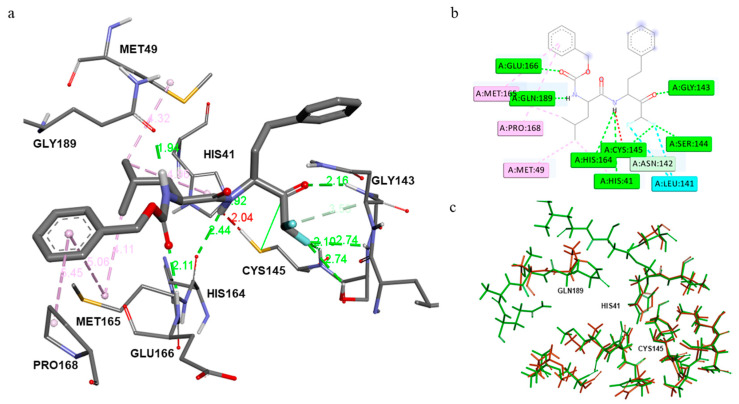
(**a**) Detailed interactions of pose 2 of Z-Leu-Homophe-CHF_2_ inside the SARS-CoV-2 M^pro^ binding site. (**b**) 2D Interaction inside the binding pocket of the protein. (**c**) Alignment of amino acids of the binding site of hCoV-229E M^pro^ (green) and SARS-CoV-2 M^pro^ (orange).

The high similarity of the binding site (Figure 7c) between the two enzymes produced comparable results in the energy of the binding and docking pose. Indeed, the free energy of binding (Table 1) showed a slightly lower value for SARS-CoV-2 M^pro^ (Δ*G* = −7.3 kcal/mol) than for hCoV-229E M^pro^ (Δ*G* = −7.0 kcal/mol).

In order to evaluate the scoring function of AutoDock 4.2, the famous N3 inhibitor was docked as the experimental data of the inhibition constant (*K*_i_), and the EC_50_ values are available, respectively for hCoV-229E M^pro^ [56] and SARS-CoV-2 M^pro^ [57]. The SARS2 experimental *K*_i_ value was not available due to the high inhibition potency. However, the results are shown in Table S1, demonstrating the high accuracy of the scoring function used by AutoDock 4.2.

By visualizing in detail the pose of Z-Leu-Homophe-CHF_2_ inside the pocket of SARS-CoV-2 M^pro^ (Figure 7a), we can see that the difluoromethyl ketone is located at a distance of 3.38 A from Cys145, optimal for a nucleophilic attack on the carbonyl group. The oxygen atom of the aldehyde group also plays a crucial role in stabilizing the inhibitor’s conformations, by forming a 2.16 Å hydrogen bond with the backbone of residue Cys143 at the S1 site. Furthermore, the amide bonds on the Z-Leu-Homophe-CHF_2_ chain form hydrogen bonds with the main chains of Cys145 (3.44 Å), Glu 166 (2.11 Å), and Gln 189 (1.94 Å), respectively. The isobutyl moiety of Z-Leu-Homophe-CHF_2_ in S2 inserts deeply into the S2 site, stacking with the imidazole ring of His 41. The side chains of Met49, Met165, Asp187, and Pro168, also surround the isobutyl group, producing extensive hydrophobic interactions (Figure 7b).

Furthermore, the difluoromethyl ketone moiety is further stabilized by the halogen interaction with Leu141 at 3.68 Å and the H-halogen bonds with Ser144 and Cys145 at 2.74 Å. During the MD simulation, the ligand is further stabilized by further water molecules bridging the residues Asn147 and Glu166 with the ester and amide groups of the ligand. The bulky benzyl group extends into the S4 site cavity, forming *π*-CH interactions with Gln189. Unlike the complex with E229, this region of the ligand is not exposed to the solvent.

A detailed analysis of the trajectories developed by MD simulation highlights the good stability of the protein-ligand complex. Z-Leu-Homophe-CHF_2_ reaches equilibrium after 20 ns, while the protein structure seems to reach a slight flexion at 65 ns, remaining, however, at low RMSD levels. The DCCM plots (See Figure S7) of the Z-Leu-Homophe-CHF_2_-SARS-CoV-2 M^pro^ complex show a correlation region in the binding site domain and anti-correlation zones in domain II and domain III, confirming the analysis of RMSFs (see Figure S7), showing fluctuations between the residues 40–70 (domain II) and 210–240 (domain III).

**Figure 8:**
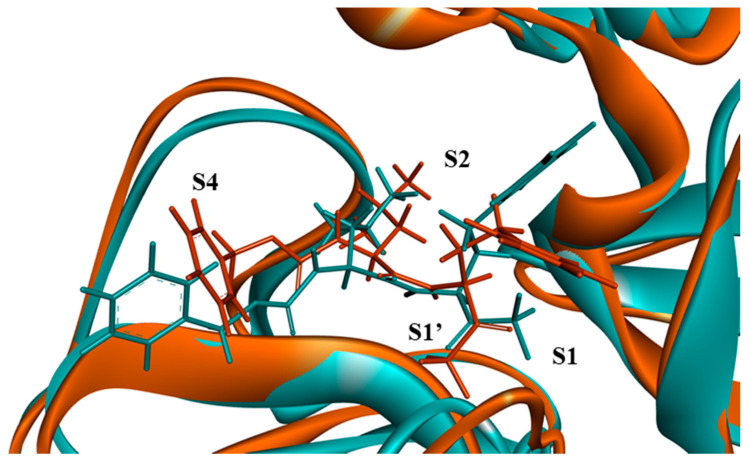
Alignment of the binding site amino acids of hCoV-229E M^pro^ (marine green) and SARS-CoV-2 M^pro^ (orange) in complex with Z-Leu-Homophe-CHF_2_, represented as a hydrophobic surface (hydrophobic: brown, hydrophilic: blue, neutral: white). Sites S1, S1’, S2, S4 of M^pro^ are indicated.

Despite the small size of the isobutyl substituent, this is possible because the S2 pocket of SARS-CoV-2 M^pro^ is flexible enough to contract and enclose small substituents. This plasticity is expressed in a conformational change of residue Gln189, both in the main and side chains. As a consequence of these changes, the side-chain oxygen of Gln189 can accept a 1.94 Å hydrogen bond from the main-chain NH residue in the Z-Leu-Homophe-CHF_2_ complex (Figure 8). However, the affinity of Z-Leu-Homophe-CHF_2_ for the S2 pocket of hCoV-229E M^pro^ is good because of an almost ideal match of size and not requiring conformational changes, which this enzyme would not be able to undergo because of the replacement of the flexible Gln189 by the more rigid proline [55]. The more remarkable plasticity of the S2 pocket can accommodate the isobutyl substituent in a deeper way. Thus, both effects could increase the stability, hence the affinity, of Z-Leu-Homophe-CHF_2_ against SARS-CoV-2 M^pro^.

## 3. Materials and Methods

### 3.1. Chemistry

#### 3.1.1. General Experimental Information

Melting points were determined on a Reichert–Kofler hot-stage microscope (Vienna, Austria) and are uncorrected. Mass spectra were obtained on a Shimadzu QP 1000 instrument (EI, 70 eV) (Shimadzu Corporation, Kyoto, Japan) and on a Brüker maXis 4G instrument (ESI-TOF, HRMS) (Brüker Corporation, Billerica, MA, USA). ^1^H, ^13^C and ^19^F NMR spectra were recorded at 297 K on a Brüker Avance III 400 spectrometer (400 MHz for 1H, 100 MHz for ^13^C, 40 MHz for 15N, 376 MHz for ^19^F), equipped with a directly detecting broadband observe (BBFO) probe, with a Brüker Avance III 500 spectrometer (500 MHz for ^1^H, 125 MHz for ^13^C) using a Prodigy cryoprobe, and with a Bruker DRX 200 spectrometer (200 MHz for ^1^H, 50 MHz for ^13^C) with a ^1^H/^13^C dual probe (Brüker Corporation, Billerica, MA, USA).

The center of the solvent signal was used as an internal standard, which was related to TMS with *δ* 4.87 ppm (^1^H in CD_3_OD), *δ* 128.06 ppm (^13^C in C_6_D_6_) and *δ* 49.00 ppm (^13^C in CD_3_OD). Absolute referencing via *Ξ* ratio was used for the ^19^F NMR spectra. Spin-spin coupling constants (*J*) are given in Hz. In nearly all cases, the full and unambiguous assignment of all resonances was performed by combined application of standard NMR techniques, such as APT, HSQC, HMBC, HSQC-TOCSY, COSY, and NOESY experiments.

All the reactions were carried out under an inert atmosphere of argon. THF was distilled over Na/benzophenone. Chemicals were purchased from Sigma-Aldrich (Sigma-Aldrich, St. Louis, Missouri, USA), Acros (Thermo Fisher Scientific, NJ, USA), Alfa Aesar (Thermo Fisher, Kandel, Germany), Fluorochem (Hadfield Derbyshire, United Kingdom) and TCI Europe (Tokyo Chemical Industry, Tokyo, Japan). Solutions were evaporated under reduced pressure with a rotary evaporator. TLC was carried out on aluminum sheets precoated with silica gel 60F254 (Merchery-Nagel, Merk, Darmstadt, Germany); the spots were visualized under UV light (λ = 254 nm).

#### 3.1.2. Synthesis of Fmoc-d-Homophe Weinreb Amide (**10**)

To a solution of Fmoc-d-Homophe-OH (1.0 g, 0.0025 mol, 1.0 equiv) in dry CPME (10 mL) was added 1,1′-carbonyldiimidazole (0.444 g, 0.0027 mol, 1.1 equiv) in one portion, and the resulting mixture was allowed to stir for 1 h at rt. Then, *N,O*-dimethylhydroxylamine hydrochloride (DMHA, 0.270 g, 0.0027 mol, 1.1 equiv) was added to the mixture, turning the solution a cloudy white. The reaction mixture was stirred at rt overnight, then quenched with 3 mL of a saturated aq. solution of NH_4_Cl, and the aqueous layer was extracted with ethyl acetate (2 × 10 mL). The combined organic layers were washed with a saturated aq. solution of NaHCO_3_ (3 × 10 mL) and brine (3 × 10 mL) then, dried over anhydrous Na_2_SO_4_ and the solvent was removed under reduced pressure to afford the corresponding Fmoc-d-Homophe Weinreb Amide **10**, in 95% yield (1.05 g), as a yellow oil without any further purification. ^1^H NMR (200 MHz, CD_3_OD) *δ*: 7.74–7.61 (m, 4H, Ar-H), 7.37–7.13 (m, 9H, Ar-H), 4.54 (m, 1H, α-CH), 4.32 (d, 2H, ^3^*J*_H,H_ = 7.17 Hz, CH_2_), 4.14 (t, 1H, ^3^*J*_H,H_ = 6.62 Hz, Fmoc-CH), 3.51 (s, 3H, OC*H*_3_), 3.09 (s, 3H, NC*H*_3_), 2.78–2.50 (m, 2H, C*H*_2_Ph), 1.98–1.87 (m, 2H, C*H*_2_-CH Homophe).^13^C NMR (50 MHz, CD_3_OD) *δ*: 174.8 (C=O Fmoc), 158.5 (C=O Homophe), 145.3 (Fmoc Ar-C), 145.0 (Fmoc Ar-C), 142.5 (Ph C-1), 129.6 (2C, Ph C-3,5), 129.4 (2C, Ph C-2,6), 128.7 (2C, Fmoc Ar-CH), 128.1 (2C, Fmoc Ar-CH), 127.0 (Ph C-4), 126.2 (Fmoc Ar-CH), 120.9 (Fmoc Ar-CH), 67.8 (CH_2_ Fmoc), 61.8 (O*C*H_3_), 51.9 (N*C*H_3_), 48.3 (CH Fmoc), 34.0 (*C*H_2_-CH Homophe), 32.9 (*C*H_2_Ph). HRMS (ESI), *m*/*z*: calcd. for C_27_H_28_N_2_O_4_Na^+^: 467.1945 [M + Na]^+^; found: 467.1941.

#### 3.1.3. Synthesis of NH_2_-Homophe Weinreb Amide (**11**)

To a solution of **10** in dry DMF (10 mL), piperidine (20%) was added and the resulting mixture was left stirring at rt overnight. The reaction mixture was dried under reduced pressure, and the crude product was purified via silica gel chromatography (DCM: MeOH 98:2) to afford compound **11** in 96% yield (0.504 g) as a yellow oil. ^1^H NMR (200 MHz, CD_3_OD) *δ*: 7.33–7.19 (m, 5H, Ph H-1,2,3,4,5,6), 3.81 (m, 1H, α-C*H* Homophe), 3.61 (s, 3H, OC*H*_3_), 3.20 (s, 3H, NC*H*_3_), 2.78–2.64 (m, 2H, C*H*_2_Ph), 2.04–1.76 (m, 2H, C*H*_2_-CH Homophe). ^13^C NMR (50 MHz, CD_3_OD) *δ*: 162.0 (C=O Homophe), 142.6 (Ph C-1), 129.6 (2C, Ph C-3,5), 129.4 (2C, Ph C-2,6), 127.0 (Ph C-4), 61.9 (O*C*H_3_), 51.1 (N*C*H_3_), 37.3 (*C*H_2_-CH Homophe), 32.7 (*C*H_2_Ph). HRMS (ESI), *m*/*z*: calcd. for C_12_H_19_N_2_O_2_^+^: 223.1446 [M + H]^+^; found: 223.1441.

#### 3.1.4. Synthesis of Z-Leu-Homophe Weinreb Amide (**12**)

Compound **11** (176 mg, 0.792 mmol, 1.2 equiv) and Z-Leu-OH (175 mg, 0.66 mmol, 1 equiv) were dissolved in dry DMF (8 mL) and EDCI (152 mg, 0.792 mmol, 1.2 equiv) and HOBt (107 mg, 0.792 mmol, 1.2 equiv) were added to the mixture at rt. The reaction was left stirring under Ar atmosphere at rt overnight. The reaction mixture was then diluted with ethyl acetate and was washed with saturated aq. solution of NH_4_Cl (3 × 10 mL), saturated aq. solution of NaHCO_3_ (3 × 10 mL) and brine (3 × 10 mL). The organic layer was dried over anhydrous Na_2_SO_4_, filtered and concentrated under reduced pressure to afford **12** as a colorless liquid which readily crystallizes in a white solid upon trituration with diethyl ether (65% yield, 202 mg). ^1^H NMR (400 MHz, CD_3_OD) *δ*: 7.36 (m, 2H, Bn H-2,6), 7.31 (m, 2H, Bn, H-3,5), 7.27 (m, 1H, Bn H-4), 7.24 (m, 2H, Ph, H-3,5), 7.18 (m, 2H, Ph H-2,6), 7.16 (m, 1H, Ph H-4), 5.13–5.09 (m, AB System, ^2^*J*_A,B_ = 12.3 Hz, 2H, C*H*_2_O), 4.77 (m, 1H, α-C*H* Homophe), 4.26 (dd, ^3^*J*_H,H_ = 9.1 Hz, ^3^*J*_H,H_ = 5.9 Hz, 1H, α-C*H* Leu), 3.57 (s, 3H, OC*H*_3_), 3.13 (s, 3H, NC*H*_3_), 2.75–2.61 (m, 2H, C*H*_2_Ph), 2.05–1.85 (m, 2H, C*H*_2_-CH Homophe), 1.72 (m, 1H, C*H*-CH_3_), 1.57 (m, 2H, C*H*_2_-CH Leu), 0.94 (d, ^3^*J*_H,H_ = 6.67 Hz, 3H, C*H*_3_-CH), 0.91 (d, ^3^*J*_H,H_ = 6.67 Hz, 3H, CH_3_-CH). ^13^C NMR (100 MHz, CD_3_OD) *δ*: 175.5 (C=O Leu), 174.1 (C=O Homophe), 158.4 (C=O CBZ), 142.2 (Ph C-1), 138.2 (Bn C-1), 129.8 (2C, Ph C-2,6), 129.5 (2C, Bn, C-3,5), 129.4 (2C, Ph C-3,5), 129.0 (Bn C-4), 128.8 (2C, Bn C-2,6), 127.1 (Ph C-4), 67.6 (*C*H_2_O), 61.9 (O*C*H_3_), 54.7 (α-C*H* Leu), 50.2 (α-C*H* Homophe), 41.9 (*C*H_2_-CH Leu), 34.1 (*C*H_2_-CH Homophe), 32.8 (*C*H_2_Ph), 32.4 (N*C*H_3_), 25.8 (*C*H-CH_3_), 23.5 (*C*H_3_-CH), 22.0 (*C*H_3_-CH). HRMS (ESI), m/z: calcd. for C_26_H_35_N_3_O_5_Na^+^: 492.2487 [M + Na]^+^; found: 492.2469.

#### 3.1.5. Synthesis of Z-Leu-Homophe-CHF_2_ (**8a**,**b**)

To a solution of compound **12** (120 mg, 0.255 mmol, 1 equiv) in dry THF (5 mL) cooled at 0 °C was added (difluoromethyl)trimethylsilane (0.18 mL, 1.275 mmol, 2 equiv) under Ar atmosphere. Then, potassium *tert*-pentoxide 0.9 M (1.25 mL, 1.122 mmol, 4.4 equiv) was added dropwise with good stirring at 0 °C during a period of 15 min. The reaction mixture was further stirred to reach rt within 3 h. After complete conversion of the starting material, the reaction mixture was quenched with saturated aqueous NH_4_Cl solution (3 mL) and extracted with ethyl acetate (3 × 5 mL). The organic layer was washed with brine (5 mL), dried over Na_2_SO_4_, filtered and concentrated under reduced pressure. The crude was purified via column chromatography on silica gel (DCM: MeOH 98:2) to afford the corresponding compounds **8a**,**b** as diastereomeric mixture (3:1) in 87% yield (106 mg). ^1^H NMR (400 MHz, CD_3_OD) *δ*: 7.34 (m, 2H, Bn H-2,6), 7.31 (m, 2H, Bn), 7.27 (m, 1H, Bn), 7.24 (m, 2H, Ph), 7.16 (m, 2H, Ph H-2,6), 7.16 (m, 1H, Ph), 5.76/5.69 (td, ^2^*J*_H,F_ = 54.4 Hz, ^3^*J*_H,H_ = 12.2 Hz, 1H, C*H*F_2_), 5.14–5.06 (m, 2H, C*H*_2_O), 4.20 (m, 2H, α-C*H* Homophe, α-C*H* Leu), 2.65–2.47 (m, 2H, C*H*_2_Ph), 2.06–1.75 (m, 2H, C*H*_2_-CH Homophe), 1.74 (m, 1H, C*H*-CH_3_), 1.57 (m, 2H, C*H*_2_-CH Leu), 0.97 (d, ^3^*J*_H,H_ = 6.7 Hz, 3H, C*H*_3_-CH), 0.94 (d, ^3^*J*_H,H_ = 6.7 Hz, 3H, C*H*_3_-CH). ^13^C NMR (100 MHz, CD_3_OD) *δ*: 175.77/175.72 (C=O Leu), 158.6/158.5 (C=O CBZ), 143.2 (Ph C-1), 138.2 (Bn C-1), 129.6 (2C, Ph C-2,6), 129.4 (2C, Ph), 129.0 (Bn), 128.9 (2C, Bn) 128.9 (2C, Bn C-2,6), 126.89/126.83 (Ph C-4), 116.1 (t, ^1^*J*_C,F_ = 247.0 Hz, *C*HF_2_), 96.8 (HO-*C*-OH), 67.73/67.68 (*C*H_2_O), 55.25/55.19 (α-C*H* Leu),53.45/53.14 (α-C*H* Homophe), 41.89/41.86 (*C*H_2_-CH Leu), 33.42/33.20 (*C*H_2_Ph), 31.93/31.56 (*C*H_2_-CH Homophe), 25.85/25.83 (*C*H-CH_3_), 23.33/23.31 (*C*H_3_-CH), 22.05/21.99 (*C*H_3_-CH). ^19^F NMR (376 MHz, CD_3_OD) *δ*: -136.4 (d, ^2^*J*_H,F_ = 54.7 Hz, CHF_2_). HRMS (ESI), m/z: calcd. for hydrated ketone C_25_H_32_F_2_N_2_O_5_Na^+^: 501.2176 [M + Na]^+^; found: 501.2171; m/z: calcd. for ketone C_25_H_30_F_2_N_2_O_4_Na^+^: 483.2065 [M + Na]^+^; found: 483.2066.

### 3.2. Biological Assays

#### 3.2.1. Cells and Viruses

Low-passage human embryonic lung fibroblasts (HELFs) were prepared and grown as monolayers in MEM supplemented with 10% FBS (Euroclone), 1 mM sodium pyruvate, 2 mM glutamine, 100 U mL^−1^ penicillin, and 100 μg/mL 1 streptomycin sulfate, as previously described [58]. MRC5 (ATCC^®^ CCL-171^™^) lung fibroblast were purchased from the American Type Culture Collection (ATCC) and cultured in Eagle’s minimum essential medium (MEM; EuroClone) supplemented with 10% fetal bovine serum (FBS, Euroclone), 2 mM glutamine, 1 mM sodium pyruvate, 100 U/mL penicillin, and 100 µg/mL streptomycin sulfate (P/S, both from Euroclone). Lung epithelial A549 (ATCC^®^ CCL-185^™^) cells were purchased from ATCC, and cultured in Dulbecco’s modified Eagle medium (DMEM; EuroClone), supplemented with 10% fetal bovine serum (FBS, Euroclone), 2 mM glutamine, 1 mM sodium pyruvate, 100 U/mL penicillin, and 100 µg/mL streptomycin sulfate (P/S, both from Euroclone). The human coronavirus 229E (ATCC^®^ VR-740^™^) was purchased from the ATCC and propagated and titrated on MRC5 cells.

#### 3.2.2. Cytotoxicity Assay

HELFs, MRC5, or A549 cells were seeded in 96-well plates (18,000 cells/well), and after 24 h, the cells were exposed to increasing concentrations of Z-Leu-Homophe-CHF_2_ (**8**) or vehicle (DMSO) as control. After 72 h of incubation, the number of viable cells was determined using the CellTiter-Glo Luminescent assay (Promega) according to the specifications of the manufacturer.

#### 3.2.3. Antiviral Assay

To evaluate the antiviral activity of different concentrations of Z-Leu-Homophe-CHF_2_ or remdesivir (MedChem Express), MRC5 cells seeded in 48-well plates (30,000 cells/well) and then treated with different concentrations of the compounds during infection with hCoV-229E at 100 PFU/well. Following virus adsorption (2 h at 37 °C), viral inocula were removed, and cells were maintained in a medium containing the corresponding compounds, and 2% FBS. Cells treated with vehicle (DMSO) and cells infected and treated with vehicle were used as mock infection control for normalization and infection control, respectively. After 72 h p.i., cell viability was measured using CellTiter-Glo assay as a surrogate measurement of the viral cytopathic effect (CPE), as previously described [52]. The mean values for Z-Leu-Homophe-CHF_2_ (**8**) and remdesivir were normalized to the mean values for the mock-infected control cells (DMSO-treated), and the concentration that produced 50% of reduced cell viability (EC_50_) was determined by GraphPad Prism software, version 7.

### 3.3. Computational

#### 3.3.1. Molecular Docking

Flexible ligand docking experiments, successfully used in our previous work [31,59,60,61,62,63,64], were performed employing AutoDock 4.2.6 software implemented in YASARA (v. 20.10.4, YASARA Biosciences GmbH, Vienna, Austria) [65,66], using the three-dimensional crystal structure of hCoV-229E M^pro^ (PDB ID: 1P9S), the three-dimensional crystal structure of SARS-CoV-2 M^pro^ in complex with an inhibitor N3 PRD_002214 (PDB ID: 6LU7), both obtained from the Protein Data Bank (PDB, http://www.rcsb.org), and the Lamarckian genetic algorithm (LGA). The covalent bond between the Cys145 residue and the crystallized ligand has been eliminated. His41 and Cys145 residues were protonated and optimized using YASARA software. The maps were generated by the program AutoGrid (4.2.6) with a spacing of 0.375 Å and dimensions that encompass all atoms extending 5 Å from the surface of the structure of the crystallized ligands. The details were reported in the SI.

#### 3.3.2. Molecular Optimization

The semiempirical calculations were performed using the parameterized model number 6 Hamiltonian [67], as implemented in MOPAC package (J.J.P. Stewart, MOPAC2016, (2017)) (MOPAC2016 v. 18.151, Stewart Computational Chemistry, Colorado Springs, CO, USA).

#### 3.3.3. Molecular Dynamics Simulations

The MD simulations of the M^pro^/ligand complexes were performed with the YASARA Structure package (19.11.5) [64]. The details were reported in the SI.

## 4. Conclusions

To sum up, in this work, we applied the chemistry of α-fluorinated organometallic methyl type carbanions carried out in our labs, to the design of a peptide-based α,α-difluoromethyl ketone, having the –COCHF_2_ moiety at the *C*-terminal of the peptidic framework as electrophilic warhead able to tackle cysteine proteases which are vital for coronaviruses’ survival. Specifically, our pseudo-peptide, with the Z-Leu-Homophe-CHF_2_ motif, was developed on the basis of a structurally related lead compound targeting SARS-CoV M^pro^ with activity in the micromolar range. Biological assays performed on cells infected with hCoV-229E, one of the four human coronaviruses associated with respiratory distress, showed that our compound exerts a remarkable antiviral activity (EC_50_ = 12.9 μM), with a shallow cytotoxicity profile evaluated in three different types of uninfected cells (A549, HELFs, and MRC5 cells). An exhaustive work of molecular docking and molecular dynamics indicated that this fluorinated pseudo-dipeptide might efficaciously bind to the intended cysteine target HCoV-229E M^pro^. Moreover, due to the high similarity between the M^pro^ of hCoV-229E and SARS-CoV-2 (the causative agent of COVID-19), we also performed an in silico analysis towards SARS-CoV-2 M^pro^. The latter study showed that Z-Leu-Homophe-CHF_2_ might bind SARS-CoV-2 M^pro^ as effectively as for hCoV-229E M^pro^, suggesting that this fluorinated pseudo-peptide may be exploited as a new reference structure for the future design of druggable compounds with activity against coronaviruses.

## Figures and Tables

**Table 1:** Calculated binding energies (kcal/mol) and *K*_i_ (μM) for the binding sites of HCoV-229E M^pro^ and SARS-CoV-2 M^pro^ for Z-Leu-Homophe-CHF_2_.

Enzyme	Calcd. Δ*G*	Calcd. *K*_i_ (µM)
hCoV-229E M^pro^	−7.0	7.3
SARS-CoV-2 M^pro^	−7.3	4.4

## Data Availability

The data that support the findings of this study are available from the corresponding authors upon reasonable request.

## Supplementary Materials

The following are available online at https://www.mdpi.com/1422-0067/22/3/1398/s1.

## References

[1] Peeri N.C., Shrestha N., Rahman M.S., Zaki R., Tan Z., Bibi S., Baghbanzadeh M., Aghamohammadi N., Zhang W., Haque U. (2020). The SARS, MERS and novel coronavirus (COVID-19) epidemics, the newest and biggest global health threats: What lessons have we learned?. Int. J. Epidemiol..

[2] Phanuphak N., Gulick R.M. (2020). HIV treatment and prevention 2019: Current standards of care. Curr. Opin. HIV AIDS.

[3] Sandmann L., Schulte B., Manns M.P., Maasoumy B. (2019). Treatment of chronic hepatitis C: Efficacy, side effects and complications. Visc. Med..

[4] Duncan J.D., Urbanowicz R.A., Tarr A.W., Ball J.K. (2020). Hepatitis C virus vaccine: Challenges and prospects. Vaccines.

[5] Crimi S., Fiorillo L., Bianchi A., D’Amico C., Amoroso G., Gorassini F., Mastroieni R., Marino S., Scoglio C., Catalano F. (2019). Herpes virus, oral clinical signs and QoL: Systematic review of recent data. Viruses.

[6] Agency E.M. (2020). Summary on Compassionate Use Remdesivir Gilead.

[7] Sun D. (2020). Remdesivir for treatment of COVID-19: Combination of pulmonary and IV administration may offer additional benefit. AAPS J..

[8] Jin Y., Yang H., Ji W., Wu W., Chen S., Zhang W., Duan G. (2020). Virology, epidemiology, pathogenesis, and control of COVID-19. Viruses.

[9] De Luca L., Ferro S., Buemi M.R., Monforte A.M., Gitto R., Schirmeister T., Maes L., Rescifina A., Micale N. (2018). Discovery of benzimidazole-based Leishmania mexicana cysteine protease CPB 2.8 Δ CTE inhibitors as potential therapeutics for leishmaniasis. Chem. Biol. Drug Des..

[10] Massai L., Messori L., Micale N., Schirmeister T., Maes L., Fregona D., Cinellu M.A., Gabbiani C. (2017). Gold compounds as cysteine protease inhibitors: Perspectives for pharmaceutical application as antiparasitic agents. BioMetals.

[11] Scala A., Micale N., Piperno A., Rescifina A., Schirmeister T., Kesselring J., Grassi G. (2016). Targeting of the leishmania Mexicana cysteine protease CPB2. 8ΔCTE by decorated fused benzo [b] thiophene scaffold. RSC Adv..

[12] Scala A., Rescifina A., Micale N., Piperno A., Schirmeister T., Maes L., Grassi G. (2018). Ensemble-based ADME–Tox profiling and virtual screening for the discovery of new inhibitors of the Leishmania mexicana cysteine protease CPB2. 8ΔCTE. Chem. Biol. Drug Des..

[13] Citarella A., Micale N. (2020). Peptidyl fluoromethyl ketones and their applications in medicinal chemistry. Molecules.

[14] O’Hagan D. (2008). Understanding organofluorine chemistry. An introduction to the C–F bond. Chem. Soc. Rev..

[15] Dobson L.S., Pattison G. (2016). Rh-Catalyzed arylation of fluorinated ketones with arylboronic acids. Chem. Commun..

[16] Linderman R.J., Jamois E.A. (1991). A semi-empirical and ab-initio analysis of fluoroketones as reactive electrophiles. J. Fluor. Chem..

[17] Pattison G. (2017). Conformational preferences of α-fluoroketones may influence their reactivity. Beilstein J. Org. Chem..

[18] Pattison G. (2018). Methods for the Synthesis of α, α-Difluoroketones. Eur. J. Org. Chem..

[19] Malquin N., Rahgoshay K., Lensen N., Chaume G., Miclet E., Brigaud T. (2019). CF_2_H as a hydrogen bond donor group for the fine tuning of peptide bond geometry with difluoromethylated pseudoprolines. Chem. Commun..

[20] Bordwell F.G. (1988). Equilibrium acidities in dimethyl sulfoxide solution. Acc. Chem. Res..

[21] Erickson J.A., McLoughlin J.I. (1995). Hydrogen bond donor properties of the difluoromethyl group. J. Org. Chem..

[22] Sessler C.D., Rahm M., Becker S., Goldberg J.M., Wang F., Lippard S.J. (2017). CF_2_H, a hydrogen bond donor. J. Am. Chem. Soc..

[23] Camerino E., Wong D.M., Tong F., Körber F., Gross A.D., Islam R., Viayna E., Mutunga J.M., Li J., Totrov M.M. (2015). Difluoromethyl ketones: Potent inhibitors of wild type and carbamate-insensitive G119S mutant Anopheles gambiae acetylcholinesterase. Bioorg. Med. Chem. Lett..

[24] Sowaileh M.F., Salyer A.E., Roy K.K., John J.P., Woods J.R., Doerksen R.J., Hockerman G.H., Colby D.A. (2018). Agonists of the γ-aminobutyric acid type B (GABA_B_) receptor derived from β-hydroxy and β-amino difluoromethyl ketones. Bioorg. Med. Chem. Lett..

[25] Govardhan C.P., Abeles R.H. (1990). Structure-activity studies of fluoroketone inhibitors of α-lytic protease and human leukocyte elastase. Arch. Biochem. Biophysc..

[26] Imperiali B., Abeles R.H. (1986). Inhibition of serine proteases by peptidyl fluoromethyl ketones. Biochemistry.

[27] Sham H.L., Wideburg N.E., Spanton S.G., Kohlbrenner W.E., Betebenner D.A., Kempf D.J., Norbeck D.W., Plattner J.J., Erickson J.W. (1991). Synthesis of (2S, 5S, 4R)-2, 5-diamino-3, 3-difluoro-1, 6-diphenylhydroxyhexane: The core unit of a potent HIV proteinase inhibitor. J. Chem. Soc. Chem. Commun..

[28] Kelly C.B., Mercadante M.A., Leadbeater N.E. (2013). Trifluoromethyl ketones: Properties, preparation, and application. Chem. Commun..

[29] Shao Y.-M., Yang W.-B., Kuo T.-H., Tsai K.-C., Lin C.-H., Yang A.-S., Liang P.-H., Wong C.-H. (2008). Design, synthesis, and evaluation of trifluoromethyl ketones as inhibitors of SARS-CoV 3CL protease. Bioorg. Med. Chem..

[30] Scala A., Piperno A., Micale N., Christ F., Debyser Z. (2018). Synthesis and anti-HIV profile of a novel tetrahydroindazolylbenzamide derivative obtained by oxazolone chemistry. ACS Med. Chem. Lett..

[31] Gentile D., Patamia V., Scala A., Sciortino M.T., Piperno A., Rescifina A. (2020). Putative inhibitors of SARS-CoV-2 main protease from a library of marine natural products: A virtual screening and molecular modeling study. Mar. Drugs.

[32] Piperno A., Cordaro M., Scala A., Iannazzo D. (2014). Recent highlights in the synthesis of anti-HCV ribonucleosides. Curr. Med. Chem..

[33] Pace V., Castoldi L., Mamuye A.D., Langer T., Holzer W. (2016). Catalysis, chemoselective addition of halomethyllithiums to functionalized isatins: A straightforward access to spiro-epoxyoxindoles. Adv. Synth. Catal..

[34] Pace V., Castoldi L., Holzer W. (2014). Chemoselective additions of chloromethyllithium carbenoid to cyclic enones: A direct access to chloromethyl allylic alcohols. Adv. Synth. Catal..

[35] Pace V., Castoldi L., Mamuye A.D., Holzer W.J.S. (2014). Homologation of isocyanates with lithium carbenoids: A straightforward access to α-halomethyl-and α, α-dihalomethylamides. Synthesis.

[36] Pace V., Castoldi L., Mazzeo E., Rui M., Langer T., Holzer W. (2017). Efficient access to all-carbon quaternary and tertiary alpha-functionalized homoallyl-type aldehydes from ketones. Angew. Chem. Int. Ed. Engl..

[37] Pace V., Castoldi L., Monticelli S., Rui M., Collina S.J.S. (2017). New perspectives in lithium carbenoid mediated homologations. Synlett.

[38] Pace V., Holzer W., Verniest G., Alcantara A.R., De Kimpe N. (2013). Chemoselective synthesis of N-substituted α-amino-α′-chloro ketones via chloromethylation of glycine-derived weinreb amides. Adv. Synth. Catal..

[39] Pace V., Murgia I., Westermayer S., Langer T., Holzer W. (2016). Highly efficient synthesis of functionalized alpha-oxyketones via Weinreb amides homologation with alpha-oxygenated organolithiums. Chem. Commun..

[40] Pace V., Castoldi L., Holzer W. (2013). Synthesis of α, β-unsaturated α’-haloketones through the chemoselective addition of halomethyllithiums to weinreb amides. J. Org. Chem..

[41] Pace V., Castoldi L., Holzer W. (2013). Addition of lithium carbenoids to isocyanates: A direct access to synthetically useful N-substituted 2-haloacetamides. Chem. Commun..

[42] Miele M., D’Orsi R., Sridharan V., Holzer W., Pace V. (2019). Highly chemoselective difluoromethylative homologation of iso(thio)cyanates: Expeditious access to unprecedented alpha, alpha-difluoro(thio)amides. Chem. Commun..

[43] Miele M., Citarella A., Micale N., Holzer W., Pace V. (2019). Direct and chemoselective synthesis of tertiary difluoroketones via weinreb amide homologation with a CHF2-carbene equivalent. ACS Org. Lett..

[44] Ielo L., Touqeer S., Roller A., Langer T., Holzer W., Pace V. (2019). Telescoped, divergent, chemoselective C1 and C1-C1 homologation of imine surrogates: Access to quaternary chloro- and halomethyl-trifluoromethyl aziridines. Angew. Chem. Int. Ed. Engl..

[45] Castoldi L., Monticelli S., Senatore R., Ielo L., Pace V. (2018). Homologation chemistry with nucleophilic alpha-substituted organometallic reagents: Chemocontrol, new concepts and (solved) challenges. Chem. Commun..

[46] Parisi G., Colella M., Monticelli S., Romanazzi G., Holzer W., Langer T., Degennaro L., Pace V., Luisi R. (2017). Exploiting a “Beast” in carbenoid chemistry: Development of a straightforward direct nucleophilic fluoromethylation strategy. J. Am. Chem. Soc..

[47] Castoldi L., Ielo L., Hoyos P., Hernáiz M.J., De Luca L., Alcántara A.R., Holzer W., Pace V. (2018). Merging lithium carbenoid homologation and enzymatic reduction: A combinative approach to the HIV-protease inhibitor nelfinavir. Tetrahedron.

[48] Lin G., Liu H.C., Wu F.C., Chen S.J. (1994). Synthesis of aryl α, α-difluoroalkyl ketones as potent inhibitors of cholesterol esterase. J. Chin. Chem. Soc..

[49] Reiter L.A., Martinelli G.J., Reeves L.A., Mitchell P.G. (2000). Difluoroketones as inhibitors of matrix metalloprotease-13. Bioorg. Med. Chem. Lett..

[50] Stewart R., Linden R.V.d. (1960). The acidity of some aromatic fluoro alcohols and ketones. Can. J. Chem..

[51] Corman V.M., Muth D., Niemeyer D., Drosten C. (2018). Hosts and sources of endemic human coronaviruses. Advances in Virus Research.

[52] Brown A.J., Wona J.J., Graham R.L., Dinnon III K.H., Sims A.C., Feng J.Y., Cihlarb T., Denison M.R., Baric R.S., Sheahan T.P. (2019). Broad spectrum antiviral remdesivir inhibits human endemic and zoonotic deltacoronaviruses with a highly divergent RNA dependent RNA polymerase. Antivir. Res..

[53] Anand K., Ziebuhr J., Wadhwani P., Mesters J.R., Hilgenfeld R. (2003). Coronavirus main proteinase (3CLpro) structure: Basis for design of anti-SARS drugs. Science.

[54] Ziebuhr J., Heusipp G., Siddell S.G. (1997). Biosynthesis, purification, and characterization of the human coronavirus 229E 3C-like proteinase. J. Virol..

[55] Zhang L., Lin D., Kusov Y., Nian Y., Ma Q., Wang J., Von Brunn A., Leyssen P., Lanko K., Neyts J. (2020). α-Ketoamides as broad-spectrum inhibitors of coronavirus and enterovirus replication: Structure-based design, synthesis, and activity assessment. J. Med. Chem..

[56] Wang F., Chen C., Tan W., Yang K., Yang H. (2016). Structure of main protease from human coronavirus NL63: Insights for wide spectrum anti-coronavirus drug design. Sci. Rep..

[57] Jin Z., Du X., Xu Y., Deng Y., Liu M., Zhao Y., Zhang B., Li X., Zhang L., Peng C. (2020). Structure of M(pro) from SARS-CoV-2 and discovery of its inhibitors. Nature.

[58] Luganini A., Caposio P., Mondini M., Landolfo S., Gribaudo G. (2008). New cell-based indicator assays for the detection of human cytomegalovirus infection and screening of inhibitors of viral immediate-early 2 protein activity. J. Appl. Microbiol..

[59] Gentile D., Fuochi V., Rescifina A., Furneri P.M. (2020). New anti SARS-Cov-2 targets for quinoline derivatives chloroquine and hydroxychloroquine. Int. J. Mol. Sci..

[60] Amata E., Dichiara M., Gentile D., Marrazzo A., Turnaturi R., Arena E., La Mantia A., Tomasello B.R., Acquaviva R., Di Giacomo C. (2020). Sigma receptor ligands carrying a nitric oxide donor nitrate moiety: Synthesis, in silico, and biological evaluation. ACS Med. Chem. Lett..

[61] Floresta G., Patamia V., Gentile D., Molteni F., Santamato A., Rescifina A., Vecchio M. (2020). Repurposing of FDA-approved drugs for treating iatrogenic botulism: A paired 3D-QSAR/docking approach. Chem. Med. Chem..

[62] Floresta G., Gentile D., Perrini G., Patamia V., Rescifina A. (2019). Computational tools in the discovery of FABP4 ligands: A statistical and molecular modeling approach. Mar. Drugs.

[63] Floresta G., Amata E., Gentile D., Romeo G., Marrazzo A., Pittalà V., Salerno L., Rescifina A. (2019). Fourfold filtered statistical/computational approach for the identification of imidazole compounds as HO-1 inhibitors from natural products. Mar. Drugs.

[64] Floresta G., Dichiara M., Gentile D., Prezzavento O., Marrazzo A., Rescifina A., Amata E. (2019). Morphing of ibogaine: A successful attempt into the search for sigma-2 receptor ligands. Int. J. Mol. Sci..

[65] Krieger E., Koraimann G., Vriend G. (2002). Increasing the precision of comparative models with YASARA NOVA—A self-parameterizing force field. Prot. Struct. Funct. Bioinform..

[66] Krieger E., Vriend G. (2014). YASARA View—Molecular graphics for all devices—From smartphones to workstations. Bioinform..

[67] Stewart J.J. (2007). Optimization of parameters for semiempirical methods V: Modification of NDDO approximations and application to 70 elements. J. Mol. Model..

