# Supplementary material for: Pseudo-Dipeptide Bearing α,α-Difluoromethyl Ketone Moiety as Electrophilic Warhead with Activity against Coronaviruses
Source: Int J Mol Sci. 2021 Jan 30;22(3):1398. doi: 10.3390/ijms22031398 (PMC7866854; doi:10.3390/ijms22031398)
Supplement: Supplementary file 1 [file ijms-22-01398-s001.pdf]

Pseudo-dipeptide Bearing α,α-Difluoromethyl Ketone Moiety as Electrophilic Warhead with Activity Against Coronaviruses

Andrea Citarella[1,2], Davide Gentile[3], Antonio Rescifina[3], Anna Piperno[1], Barbara Mognetti[4], Giorgio Gribaudo[4], Maria Teresa Sciortino[1], Wolfgang Holzer[2], Vittorio Pace[2,5]* and Nicola Micale[1]*

[1] Department of Chemical, Biological, Pharmaceutical, and Environmental Sciences, University of Messina, V.le F. Stagno d'Alcontres 31, 98166 Messina, Italy; acitarella@unime.it (A.C.); mtsciortino@unime.it (M.T.S.); apiperno@unime.it (A.P.); nmicale@unime.it (N.M.)

[2] Department of Pharmaceutical Chemistry, University of Vienna, Althanstrasse 14, A-1090 Vienna, Austria; vittorio.pace@univie.ac.at (V.P.); wolfgang.holzer@univie.ac.at (W.H.)

[3] Department of Drug Sciences, University of Catania, V.le A. Doria, 95125 Catania, Italy; davide.gentile@phd.unict.it (D.G.); arescifina@unict.it (A.R.)

[4] Department of Life Sciences and Systems Biology, University of Torino, Via Accademia Albertina 13 10123 Torino – Italy; giorgio.gribaudo@unito.it (G.G.); barbara.mognetti@unito.it (B.M)

[5] Department of Chemistry, University of Torino, Via P. Giuria 7, 10125 Torino, Italy; vittorio.pace@unito.it

* Correspondence: nmicale@unime.it (Nicola Micale); +39 090 6766419; vittorio.pace@univie.ac.at (Vittorio Pace).

SUPPLEMENTARY MATERIALS

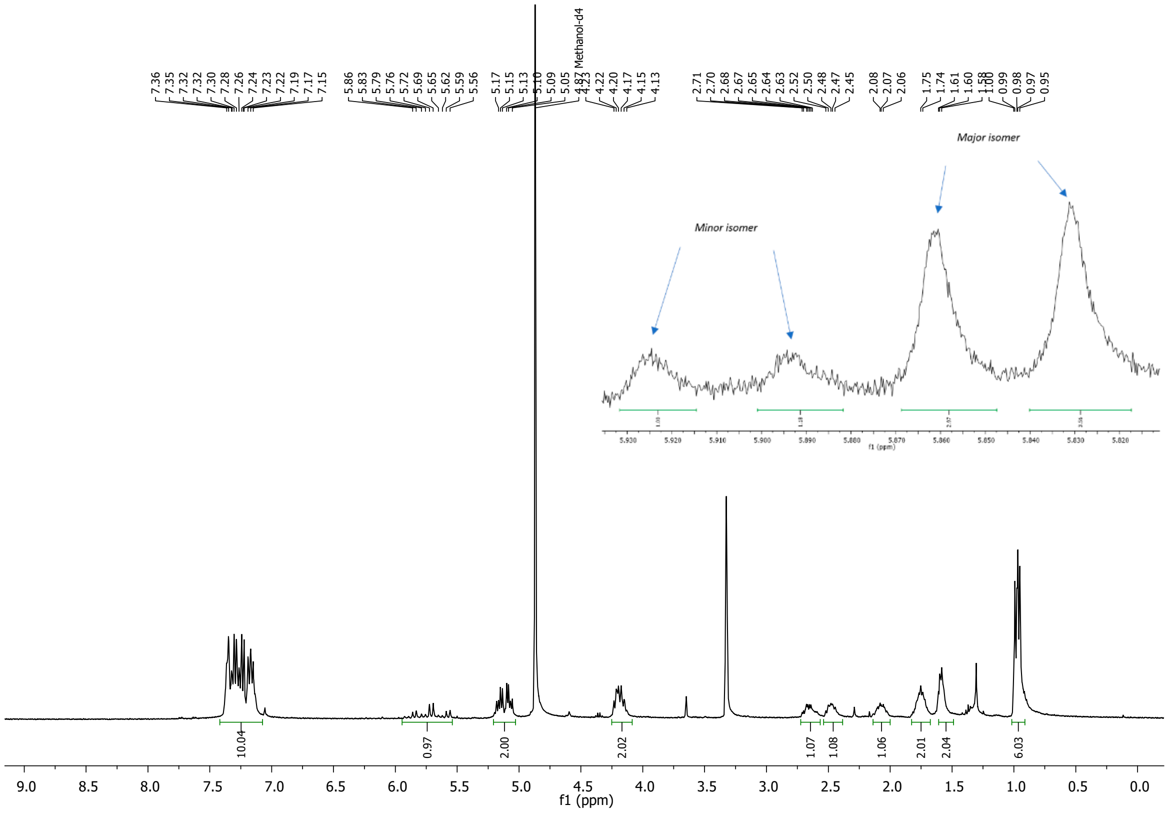

Figure S1. ^{1}H NMR spectrum of Z-Leu-Homophe-CHF_2 recorded in methanol-d_4.

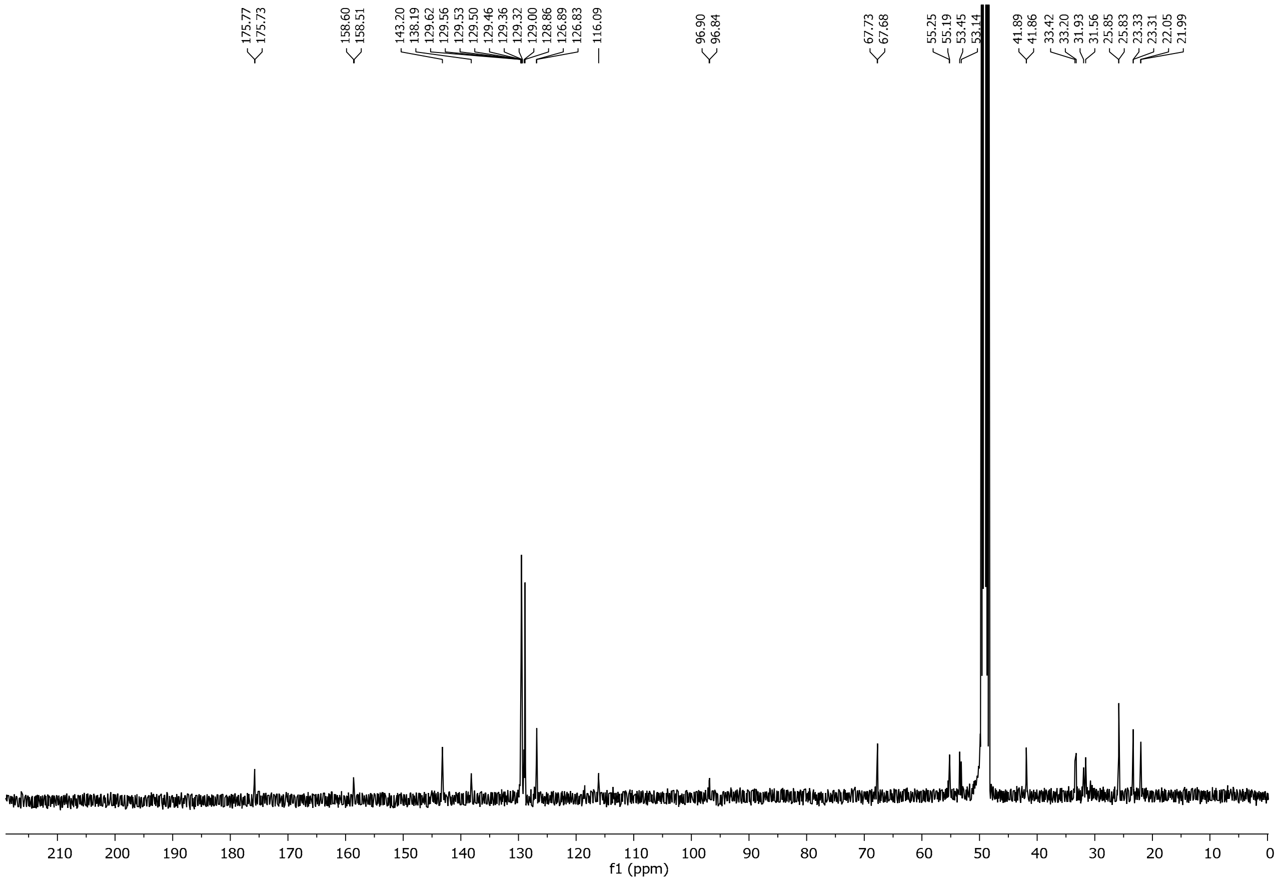

Figure S2. ^{13}C NMR spectrum of Z-Leu-Homophe-CHF_2 recorded in methanol-d_4.

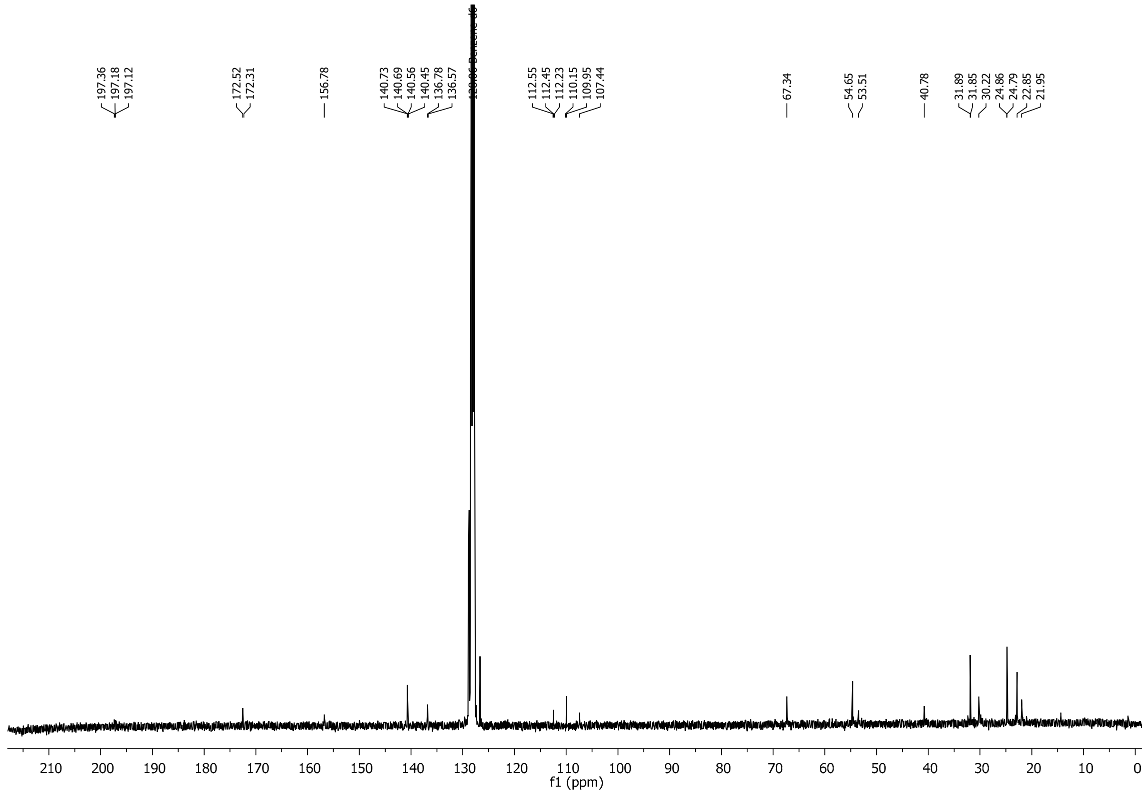

Figure S3. ^{13}C NMR spectrum of Z-Leu-Homophe-CHF_2 recorded in benzene-d_6.

a)

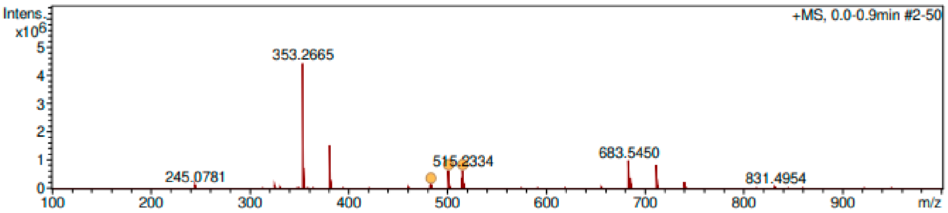

Meas. m/z	#	Ion Formula	m/z	err [ppm]	mSigma	# mSigma	Score	rdb	e⁻ Conf	N-Rule
483.2065	1	C33H27N2O2	483.2067	0.4	44.6	1	100.00	21.5	even	ok
	1	C25H30F2N2NaO4	483.2066	0.1	0.4	1	100.00	10.5	even	ok
501.2176	1	C30H30FN2O4	501.2184	1.6	33.6	1	100.00	16.5	even	ok
	2	C33H29N2O3	501.2173	-0.7	50.2	2	78.12	20.5	even	ok
	1	C25H32F2N2NaO5	501.2171	-0.9	6.5	1	100.00	9.5	even	ok
515.2334	1	C31H32FN2O4	515.2341	1.2	32.6	1	100.00	16.5	even	ok
	2	C34H31N2O3	515.2329	-1.0	49.2	2	67.72	20.5	even	ok
	1	C26H34F2N2NaO5	515.2328	-1.2	4.8	1	100.00	9.5	even	ok

b)

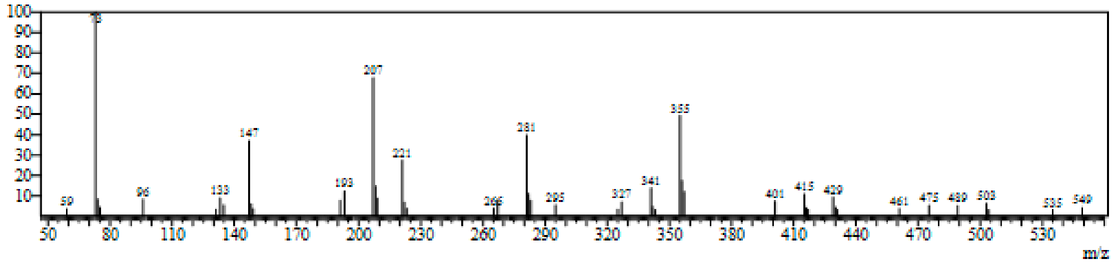

Figure S4. HRMS (a) and GC-MS (b) spectra of Z-Leu-Homophe-CHF2.

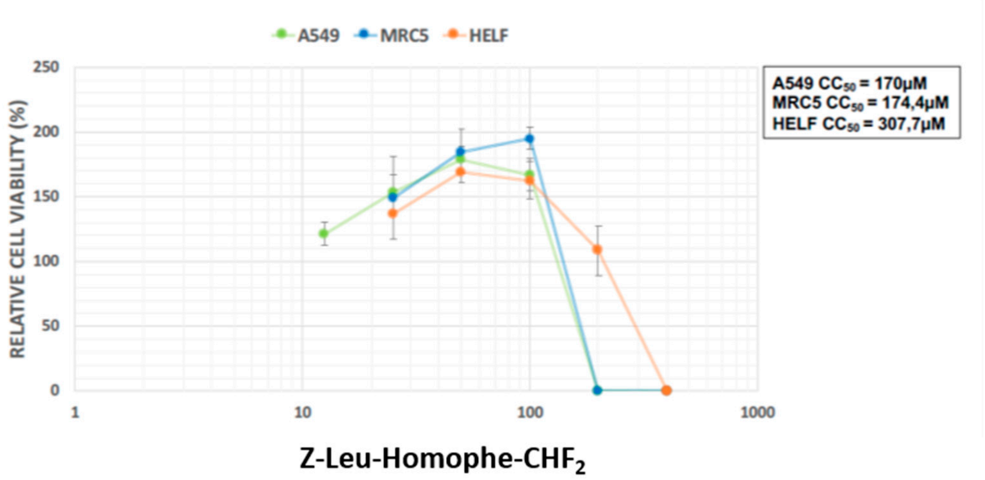

Figure S5. Effect of Z-Leu-Homophe-CHF2 on cell viability**.** A549, HELF, or MRC5 cells were treated with increasing concentrations of Z-Leu-Homophe-CHF2 or vehicle (DMSO), as control. After 72 h of incubation, cell viability was determined using the CellTiter-Glo Luminescent assay.

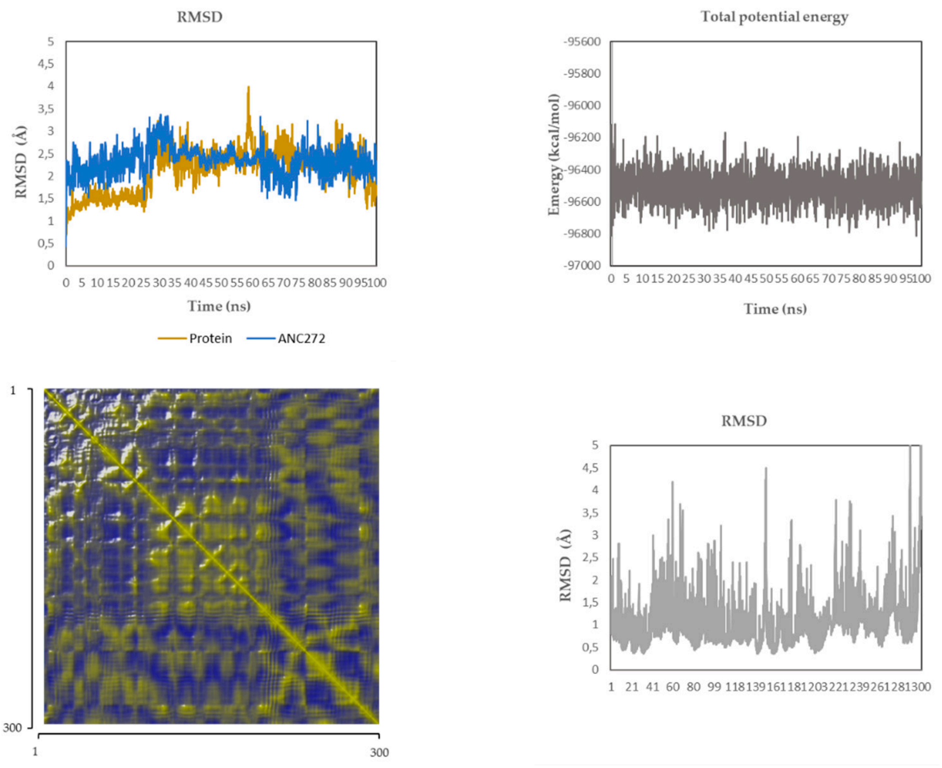

Figure S6. RMSDs of hCoV 229E M^{pro} and its complexes with Z-Leu-Homophe-CHF_2 (up-left) and Total potential energy (up-right). DCCM is visualized with colors ranging from blue (−1, fully anti-correlated) to yellow (+1, fully correlated). (down-left) Root Mean Square Fluctuation [vertical axis] for solute protein residue [horizontal axis] calculated from the average RMSF of the atoms.

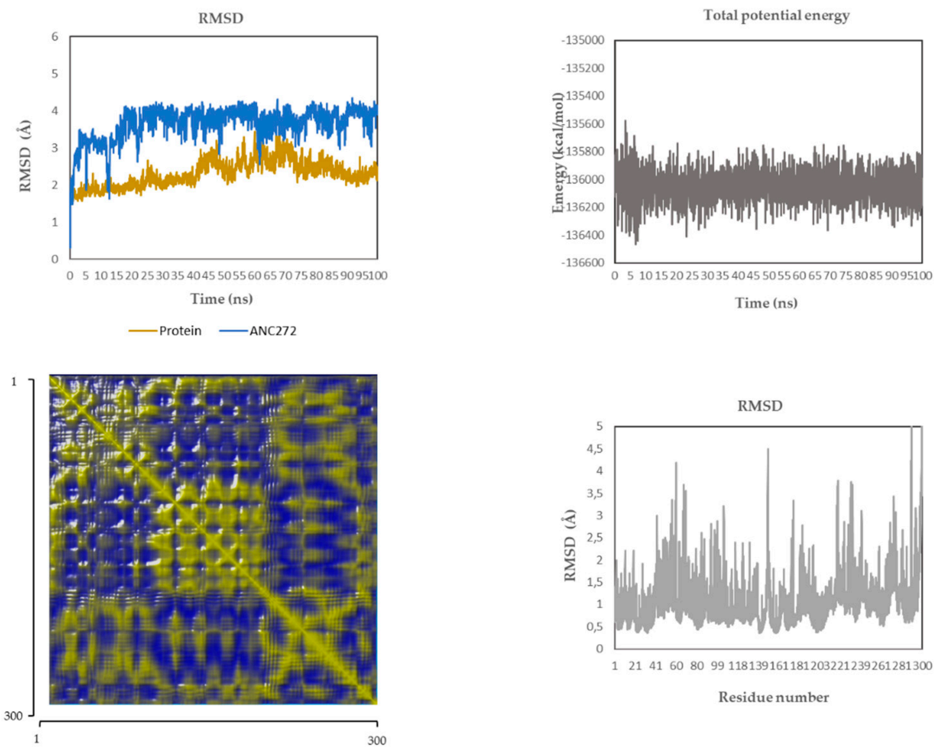

Figure S7. RMSDs of SARS-CoV-2 M^{pro} and its complexes with Z-Leu-Homophe-CHF_2 (up-left) and Total potential energy (up-right). DCCM is visualized with colors ranging from blue (−1, fully anti-correlated) to yellow (+1, fully correlated). (down-left) Root Mean Square Fluctuation [vertical axis] for solute protein residue [horizontal axis] calculated from the average RMSF of the atoms.

Table S1. Calculated energies of binding (kcal/mol) and K_i (μM), experimental *Ki,* and EC_{50} for the N3 inhibitor at the binding sites of HCoV-229E M^{pro} and SARS-CoV-2 M^{pro}.

Enzyme	Calcd. Δ*G*	Calcd. K_i (μM)	Exp. K_i (μM)	Exp. EC_{50} (μM)
hCoV-229E M^{pro}	−8.0	1.7	1.67+-0.18	—
SARS-CoV-2 M^{pro}	−11.0	0.009	–	16.77 ±1.70

Details for the LGA molecular docking procedure

All the parameters were inserted at their default settings, as previously reported. In the docking tab, the macromolecule and ligand are selected, and LGA parameters set as ga_runs = 100, ga_pop_size = 150, ga_num_evals = 25000000, ga_num_generations = 27000, ga_elitism = 1, ga_mutation_rate = 0.02, ga_crossover_rate = 0.8, ga_crossover_mode = two points, ga_cauchy_alpha = 0.0, ga_cauchy_beta = 1.0, number of generations for picking worst individual = 10. Since no water molecules are directly involved in complex stabilization, they were not considered in the docking process. All protein amino acid residues were kept rigid, whereas all single bonds of ligands were treated as fully flexible.

Details for the molecular dynamics simulations

A periodic simulation cell with boundaries extending 8 Å from the surface of the complex was employed. The box was filled with water, with a maximum sum of all bumps water of 1.0 Å and a density of 0.997 g/mL with explicit solvent. YASARA's pK_a utility was used to assign pK_a values at pH 7.4, and system charges were neutralized with NaCl (0.9% by mass). Water molecules were deleted to readjust the solvent density to 0.997 g/mL. The final system dimensions were approximately 80 × 80 × 80 $Å^3$. The ligand force field parameters were generated with the AutoSMILES utility, employs semiempirical AM1 geometry optimization. Moreover, the assignment of charges, by the assignment of the AM1BCC atom and bond types with refinement using the RESP charges, and finally, the assignments of general AMBER force field atom types. Optimization of the hydrogen bonds network of the various enzyme-ligand complexes was obtained using the method established by Hooft *et al.* This model allowed us to address ambiguities arising from multiple side-chain conformations and protonation states that are not well resolved in the electron density. A short MD simulation was run on the solvent only. The entire system was then energy minimized using first a steepest descent minimization to remove conformational stress, followed by a simulated annealing minimization until convergence (<0.01 kcal/mol Å). The MD simulation was then initiated, using the NVT ensemble at 298 K and integration time steps for intramolecular and intermolecular forces every 1.25 fs and 2.5 fs, respectively. Finally, short 5 ns MD simulations were conducted for the assessment of the correct pose, and a final MD simulation of 100 ns was performed. The conformations of each system were recorded every 100 ps.
